# Transforming Cancer Care with Oncosomes: Insight into Biogenesis, Functional Role, and Therapeutic Potential

**DOI:** 10.3390/pharmaceutics18020207

**Published:** 2026-02-05

**Authors:** Popat Mohite, Rajesh Bogati, Aishwarya Gorad, Abhijeet Puri, Sudarshan Singh, Chuda Chittasupho

**Affiliations:** 1 AET’s St. John Institute of Pharmacy and Research, Palghar 401404, Maharashtra, India; mohitepb@gmail.com (P.M.); rajeshbogati76@gmail.com (R.B.); ashu25gorad@gmail.com (A.G.); abhijeetp@sjipr.edu.in (A.P.); 2 Office of Research Administration, Chiang Mai University, Chiang Mai 50200, Thailand; sudarshan.s@cmu.ac.th; 3 Faculty of Pharmacy, Chiang Mai University, Chiang Mai 50200, Thailand

**Keywords:** cancer cells, oncosomes, biogenesis, targeted drug delivery, therapeutic applications

## Abstract

Oncosomes, a distinct subclass of extracellular vesicles released predominantly by tumor cells, have attracted increasing interest as potential carriers for targeted drug delivery in cancer research. Characterized by their large size (1–10 µm) and complex molecular cargo, including oncogenic proteins, nucleic acids, and lipids, oncosomes provide a biologically relevant platform for investigating tumor-associated communication and cargo transport. Preclinical studies suggest that oncosomes may enable tumor-associated delivery of therapeutic agents; however, evidence to date remains largely proof-of-concept and derived from in vitro and animal models. This review summarizes current knowledge on oncosome biogenesis and molecular composition; discusses their roles in cancer progression and metastasis; and critically evaluates existing methodologies for oncosome isolation, characterization, and cargo loading, including incubation, electroporation, sonication, freeze–thaw cycling, and transfection. Potential advantages such as cargo capacity and biological compatibility are discussed alongside key challenges, including vesicle heterogeneity, limited loading efficiency, large-scale manufacturing constraints, safety considerations, and regulatory uncertainty. Future perspectives focus on addressing these technical and translational barriers to support the systematic evaluation of engineered oncosomes as an experimental platform for personalized and precision-oriented cancer research.

## 1. Introduction

Cancer is one of the leading causes of death worldwide, placing a significant burden on healthcare systems. According to the 2023 report of the World Health Organization (WHO), approximately 20 million new cancer cases and nearly 10 million cancer-related deaths occurred worldwide in 2022. After cardiovascular illnesses, cancer is the second biggest cause of death globally. The Global Cancer Observatory (GLOBOCAN) 2022 estimates indicated that lung cancer was the most frequently diagnosed cancer, with about 2.5 million new cases, or one in eight cancers worldwide (12.4% of all cancers). It was followed by female breast (11.6%), colorectal (9.6%), prostate (7.3%), and stomach (4.9%) cancers [1]. Despite advances in radiotherapy, chemotherapy, and surgical interventions, the prognosis for several metastatic and aggressive cancers remains poor. The success of conventional therapies is limited by drug resistance, poor bioavailability, off-target toxicity, and tumor heterogeneity, underscoring the need for innovative, targeted therapies such as oncosome-based drug delivery [2,3,4]. The integration of nanotechnology into medicine has revolutionized drug-delivery systems (DDSs) by enabling precision and targeted therapy, which is crucial for cancer treatment. Among the numerous nano-carriers under investigation, extracellular vesicles (EVs) have gained significant attention in recent years due to their natural role in intracellular communication and their capacity to transport bioactive molecules. Compared with well-established EV subclasses such as exosomes and microvesicles, oncosomes are currently emerging as a promising approach for targeted drug delivery in oncology. They are large, membrane-bound vesicles, ranging from 1 to 10 μm in diameter, and are primarily released by cancer cells. Oncosomes are the largest and most varied vesicles, measuring 1–10 µm. They have irregular or varied shapes. Microvesicles are smaller, ranging from about 100 to 1000 nm, and are usually spherical to oval. Exosomes are the smallest, measuring approximately 30–150 nm. They have a more uniform, spherical shape, which helps them remain stable and reliable for drug delivery. Oncosomes have a high drug-loading capacity and can target tumors effectively, but they face challenges due to their significant variability and safety concerns. In contrast, exosomes are the most studied vesicles for drug delivery because of their stability and compatibility with the body, though they have limited cargo capacity. Microvesicles strike a balance, offering greater flexibility in loading than exosomes but lacking their consistency and precision in targeting [5]. Oncosomes are carriers of oncogenic proteins, such as mutant epidermal growth factor receptor variant III (EGFRvIII), which can alter recipient cell behavior and contribute to tumor progression. They are distinct from other EVs not only in their size but also in their capacity to carry distinct cargo, including oncogenic proteins, nucleic acids, and lipids that reflect their malignant origin [6]. Additionally, oncosomes have potential for drug delivery owing to their intrinsic properties, including high biocompatibility, inherent targeting capability, and the ability to cross biological barriers. These characteristics enable them to deliver therapeutic agents directly to tumour sites, improving therapeutic efficiency and minimizing unwanted systemic toxicity. Recent investigations have demonstrated their use as carriers for chemotherapeutic agents, small interfering RNAs (siRNAs), and other bioactive molecules, highlighting their role in enhancing drug-targeting precision and overcoming resistance mechanisms frequently encountered during cancer treatment [7,8]. However, despite the promises, oncosomes in clinical practice face notable challenges. Major issues include difficulties achieving consistent large-scale production, heterogeneity in vesicle size, and the absence of standardized protocols for isolation and characterization. Moreover, detailed insights into the biodistribution, pharmacokinetics, and potential immunogenicity of oncosome-based therapeutics are critical for successful translation from bench to bedside. This review aims to provide an in-depth analysis of oncosomes as DDS, including their biogenesis, molecular composition, methods of isolation and characterization, existing challenges, and potential future use in personalized cancer therapy [8,9].

## 2. Biogenesis of Oncosomes

To overcome the limitations of conventional cancer therapies, nanotechnology-based drug delivery systems have emerged as an effective platform. Cancer treatments are limited by drug resistance, systemic toxicity, poor solubility, and lack of tumor specificity. To address these challenges, various nanocarrier systems have been developed to enhance the targeted delivery of therapeutic agents to tumor tissues while minimizing damage to healthy cells [3,10]. Nanocarriers are submicron vectors, ranging from 1 to 1000 nm that can encapsulate and transport drugs, nucleic acids, or imaging agents. They protect therapeutic agents from degradation and enable sustained, controlled release, passive and active tumor targeting, and improved therapeutic efficacy [11,12]. Nanocarriers include prominent systems such as liposomes, polymeric nanoparticles, dendrimers, micelles, inorganic nanoparticles, and vesicular systems, each with unique structures and functions. Vesicular systems, which are made up of one or more lipid or amphiphilic bilayers surrounding an aqueous core, are a class of nanocarriers that resemble biological membranes. They are particularly useful for drug delivery due to their biocompatibility, ability to encapsulate a range of molecules, and potential for membrane fusion with target cells. Exosomes, microvesicles, apoptotic bodies, and large oncosomes are among the subtypes of biologically derived extracellular vesicles, which are broadly categorized by size, biogenesis, and functional roles [13]. Exosomes are small vesicles with a diameter of 30 to 150 nm that originate from the inward budding of multivesicular bodies (MVBs) within the endosomal system and are released via exocytosis. They carry tetraspanins (CD9, CD63, CD81), nucleic acids, and lipids reflective of their cell of origin. Due to their stability, ability to cross biological barriers, and low immunogenicity, exosomes have been explored for drug and Ribonucleoside (RNA) delivery in cancer and neurological disorders [14]. Direct outward budding of the plasma membrane larger and usually range in size from 100 to 1000 nm produces microvesicles (MVs), which are slightly larger and typically range from 100 to 1000 nm. They transport a wide range of materials, such as DNA, RNA, cytosolic proteins, and membrane receptors. Microvesicles have attracted interest as gene and drug delivery vehicles and play important roles in coagulation, immunological regulation, and cancer development [15]. Even larger apoptotic bodies (500–2000 nm) are released when a cell undergoes programmed cell death. They primarily assist phagocytes in removing dying cells by encapsulating nuclear fragments, organelles, and other cellular debris. Apoptotic bodies have been linked to immunological regulation and autoimmunity, although their therapeutic potential remains less well characterized [16]. Oncosomes are a distinct subtype of EVs, classified based on their relative sizes, ranging from small oncosomes (≈100–400 nm) to large oncosomes (1–10 µm). They are primarily released from tumor cells through the plasma membrane’s outward budding. These vesicles primarily contain nucleic acids, such as DNA, RNA, and noncoding RNAs. Additionally, the lipid composition is directly proportional to phosphatidylserine exposure and flipping, as well as to increased cholesterol content. Protein cargo in oncosomes includes ARF6, matrix metalloproteinases (MMPs), annexin A1 and A2, CK18, GAPDH, and various oncogenic protein complexes. Activation of epidermal growth factor receptor (EGFR) and AKT signaling pathways, and downregulation of DIAPH3 via the EKR pathways, are responsible for the production and release of oncosomes. Functionally, oncosomes play a t role in tumor development, growth, and metastasis by transferring oncogenic signals and biomolecules to other cells within the tumor microenvironment. This transfer contributes to tumor progression and malignancy. Key markers used to identify oncosomes include ARF6, CK18, GAPDH, MMP, Annexin A1, Annexin A2, and oncogenic protein complexes [17].

**Figure 1:**
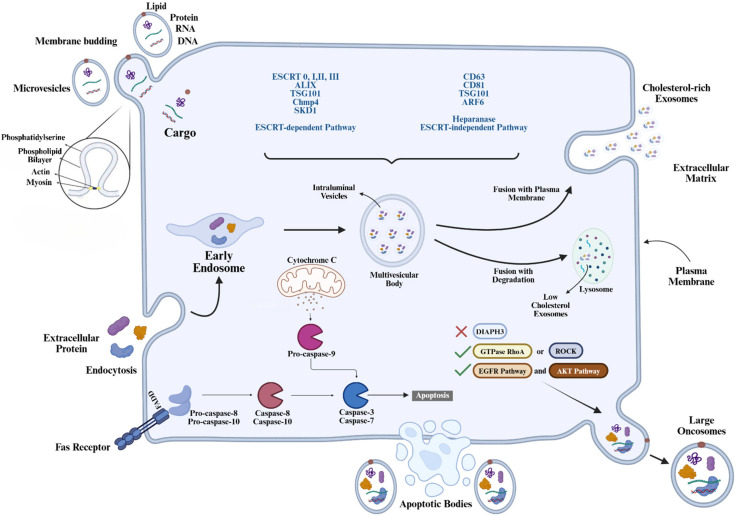
Biogenesis of Oncosomes (Created in BioRender. Bogati, R. (2026) https://BioRender.com/v1kv4l0, accessed on 24 January 2026).

The formation of large oncosomes (Figure 1) involves the shedding of substantial portions of the plasma membrane from tumor cells [18]. This process is distinct from the formation of smaller EVs, such as exosomes and microvesicles. Key factors influencing the biogenesis of Large Oncosomes include Cytoskeletal Regulation; loss of the cytoskeletal regulator diaphanous-related formin-3 (DIAPH3) promotes the formation and release of oncosomes. This loss induces a transition to an amoeboid phenotype, characterized by rapid and invasive cell movement [19]. Overexpression of oncoproteins such as caveolin-1 (CAV-1), heparin-binding epidermal growth factor (HB-EGF), and myristoylated Akt1 (MyrAkt1) can trigger the formation of LOs [18]. Activation of signaling pathways, including the EGFR and AKT1 pathways, has been associated with the induction of LO formation [20].

## 3. Release Mechanisms of Large Oncosomes from Tumor Cells

### 3.1. Plasma Membrane Blebbing and Shedding

Oncosomes are formed by the outward blebbing and scission of the plasma membrane of tumor cells. Unlike apoptotic blebs, oncosomal blebbing is a non-apoptotic event, driven by active cellular processes. This shedding is particularly enhanced in highly motile and invasive tumor cells, such as metastatic prostate cancer cells [21]. Large oncosomes originate from bulbous, amoeboid protrusions on the membrane, an effect closely associated with the amoeboid migration of transformed cells [22].

### 3.2. Cytoskeletal Remodeling

The actin cytoskeleton plays a critical role in oncosome biogenesis. Disruption of actin polymerization leads to membrane instability and promotes bleb formation. Loss of DIAPH3 (diaphanous-related formin-3), a key cytoskeletal regulator, results in the depolymerization of actin filaments and enhances the release of large oncosomes [22]. Overexpression of oncoproteins such as caveolin-1 (CAV-1), heparin-binding EGF (HB-EGF), and myristoylated AKT1 further enhances vesicle release. Additionally, ARF6, a small GTPase, is involved in both actin remodeling and the targeting of miRNA machinery into oncosomes [19]. These changes enable the dynamic reshaping of the plasma membrane, allowing large vesicles to bud off into the extracellular space.

### 3.3. Oncogenic Signaling Activation

Tumor cells with hyperactivated oncogenic pathways demonstrate increased oncosome shedding. EGFR (Epidermal Growth Factor Receptor) and AKT1 (Protein Kinase B) activation lead to cellular transformation and membrane instability, which are conducive to oncosome formation. Oncogenes like caveolin-1 (CAV-1), heparin-binding EGF-like growth factor (HB-EGF), and MyrAkt1 (myristoylated AKT1) have been shown to induce vesicle shedding when overexpressed [18]. These signals stimulate structural changes in the plasma membrane and cytoskeleton, thereby reinforcing blebbing.

### 3.4. Calcium Influx and Stimuli-Induced Shedding

**Figure 2:**
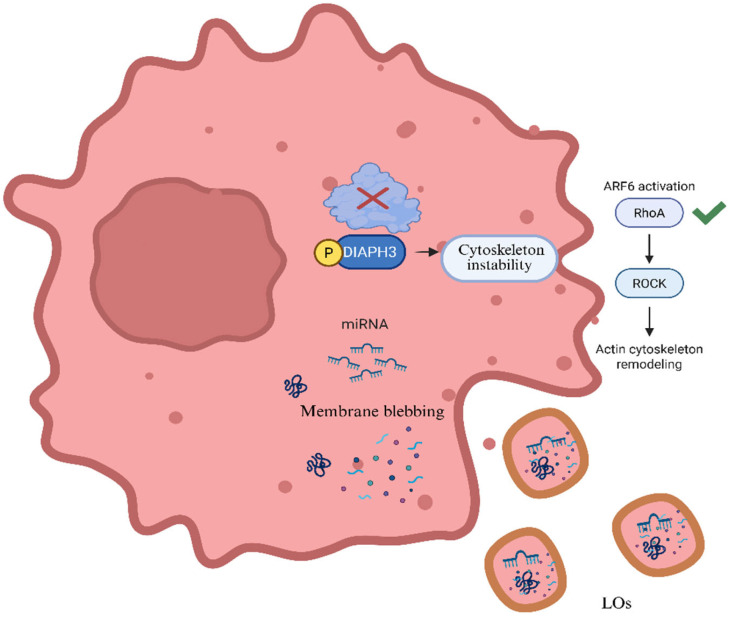
Release mechanism of oncosomes from tumor cells (Created in BioRender. Bogati, R. (2026) https://BioRender.com/dichr91, accessed on 24 January 2026).

Environmental and intracellular stimuli, such as oxidative stress, hypoxia, or cytokines, can induce the release of oncosomes by elevating intracellular calcium levels, which disrupts cytoskeletal anchoring. Triggering cytoskeletal contraction and loosening of membrane-matrix connections [18]. These calcium-dependent pathways are essential for the final scission of the bleb from the membrane. A release mechanism of oncosomes from tumor cells is demonstrated in Figure 2, whereas Table 1 presents molecular components of large oncosomes (LOs) and their functions.

## 4. Role of Oncosomes in Cancer Progression

**Figure 3:**
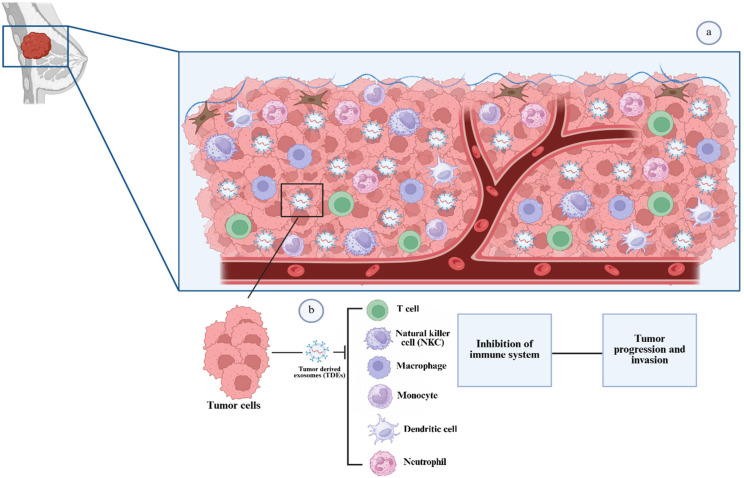
(**a**) Illustration showing tumor microenvironment interaction showing heterogeneous tumor cells surrounded by stromal components, immune cells, and blood vessels. (**b**) Mechanism of immune modulation by tumor-derived exosomes (Created in BioRender. Bogati, R. (2026) https://BioRender.com/7jl17jh, accessed on 24 January 2026).

Numerous immune cell types, including T cells, natural killer (NK) cells, macrophages, monocytes, dendritic cells, and neutrophils, interact with tumor cells (Figure 3a). These interactions are crucial because they can either support the body’s defense against the cancer or act as a catalyst for tumor development and disease progression. Tumor-derived exosomes (TDEs) play a very important role in influencing the immune responses by delivering signals to immune cells, downregulating their cytotoxic functions, aiding survival and expansion of tumor cells [30] (Figure 3b). For instance, TDEs have been shown to support tumor proliferation by increasing the availability of nutrients through interaction with the metabolic pathways of surrounding cells [31]. Additionally, TDE can modulate the behavior of stromal cells, such as macrophages, within the tumor microenvironment by inducing polarization toward the M2 phenotype, which is associated with immune suppression and tumor progression. This polarization is often mediated by miR-25-3p and miR-130b-3p, which are specific microRNAs contained within the exosome that target pathways like PTEN/PI3K/Akt to promote M2 macrophage differentiation [32].

### Metastasis

**Figure 4:**
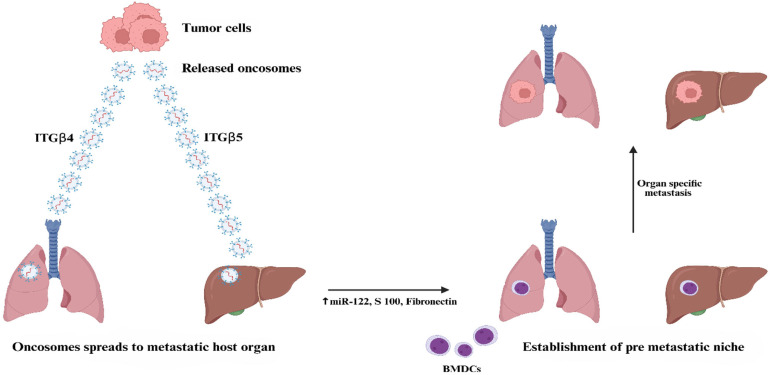
Role of oncosomes in metastasis (Created in BioRender. Bogati, R. (2026) https://BioRender.com/jviuhvf, accessed on 24 January 2026).

Figure 4 illustrates the important role of TDEs in promoting organ-specific metastasis by establishing a favorable premetastatic microenvironment. The destination of these TDEs is largely determined by the integrins present on their surface. For instance, integrin β4 (ITGβ4) is associated with directing TDE to the lungs, whereas integrin β5 (ITGβ5) is responsible for liver targeting. Upon reaching their target organs, TDEs trigger molecular and cellular changes that promote metastasis. They attract bone marrow-derived progenitor cells (BMDCs), which promote angiogenesis, tissue remodeling, and increased tumor cell invasiveness. Moreover, these TDEs increase vascular permeability, thereby allowing tumor cells to escape into the surrounding tissue. They also transport molecules, including miR-122, S100 proteins, and fibronectin, that alter nutrient availability and weaken natural defense barriers in the local environment, thereby favoring tumor growth. This mechanism explains how TDEs enable cancer cells to colonize distant organs and prepare them for metastatic expansion selectively [33]. Characteristics and Functions of Oncosomes are presented in Table 2.

## 5. Oncosomes as Drug Delivery Vehicles

Oncosomes, a subtype of EVs released by tumor cells, are being explored as natural carriers for targeted drug delivery in cancer therapy. Their origin from tumor cells enables them to home naturally to cancerous tissues, enhancing the specificity of therapeutic delivery and minimizing off-target effects. These vesicles can encapsulate various therapeutic agents, including chemotherapeutic drugs, RNA molecules, and proteins, protecting them from degradation and facilitating efficient uptake by target cells [36]. For instance, a study conducted by Dirk P. Dittmer et al. demonstrated that the tumor-derived EVs loaded with chemotherapeutic agents underwent functional reprogramming from pro-tumorigenic to an anti-tumorigenic phenotype in vivo [37]. Techniques such as electroporation and co-incubation are employed to load these vesicles with therapeutic cargo. for advancing precision medicine in oncology [38].

### 5.1. Advantages of Oncosome-Based Drug Delivery

*Biocompatibility:* Oncosomes, derived from human cells, naturally exhibit biocompatibility characteristics such as having low immunogenicity and toxicity when used in DDS [39,40,41].

*Enhanced stability:* Encapsulation of therapeutic agents into the lipid bilayer structure of oncosomes, making them safe from enzymatic degradation and improving their stability in the bloodstream [40].

*Precision targeting ability:* The oncosome inherently interacts with tumor cells due to its surface markers, allowing for more efficient delivery of therapeutic agents to cancerous cells and reducing harm to healthy cells [42].

*Capacity of diverse therapeutic drug loading:* These vesicles transport a range of molecules, including chemotherapeutic agents, genetic material, and small-molecule inhibitors, making them ideal for combination therapies [43].

### 5.2. Drug Loading and Targeting Strategies

The main challenge associated with EVs is their lower drug-loading efficiency relative to other nanoparticles, owing to their smaller size. To overcome this shortcoming, efficient drug-loading strategies have been implemented (Table 3).

## 6. Methods for Drug Loading

**Figure 5:**
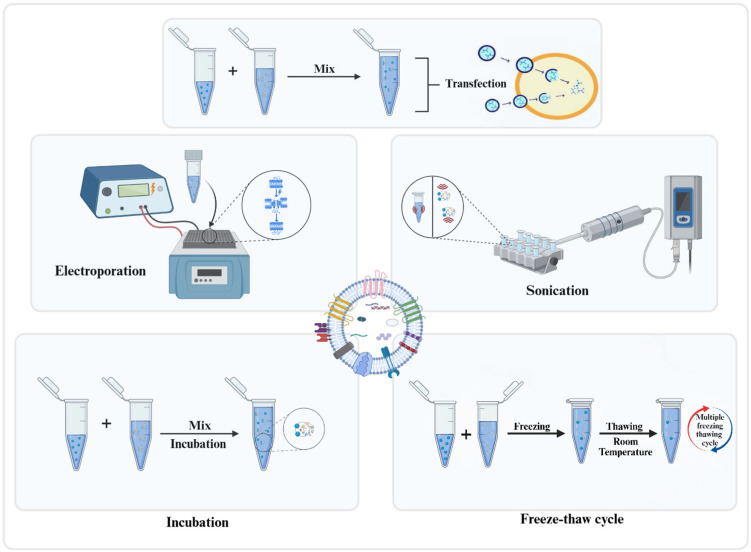
Illustration showing fabrication techniques of oncosomes (Created in BioRender. Bogati, R. (2026) https://BioRender.com/eel6673, accessed on 24 January 2026).

Drug loading methods play a critical role in determining the efficiency, stability, and therapeutic potential of oncosome-based delivery systems. Techniques such as incubation, electroporation, sonication, freeze–thaw cycling, and cell transfection are used to incorporate drugs or genetic material into oncosomes. Choosing the right method affects drug encapsulation, release profile, and vesicle integrity. Active methods typically offer higher loading efficiency, while passive techniques better preserve biological properties. Standardizing these approaches is essential for reliable, clinically applicable oncosome therapies. The different fabrication techniques of oncosomes are presented in Figure 5.

***Incubation:*** incubation methods involve co-incubating EVs with small molecules for a defined period, enabling drug loading without compromising the integrity of oncosomes. Compounds having lower molecular weight and low to medium lipophilicity, such as catalase, are easily incorporated into the vesicles [50]. For example, Renfei Wang et al. used RGD peptide to modify HEK-293 T-derived EVs, and incubated them with DOX to achieve drug loading. Incubated EVs were then radiolabeled using radioiodine-131 (^131^I), which confirmed their ability to target anaplastic thyroid carcinoma cells selectively. This targeted delivery, facilitated by the RGD peptide, demonstrates an approach for anticancer therapy using precision chemotherapy [51]. Additionally, Faruque et al. engineered EVs derived from human pancreatic ductal carcinoma cells by sequentially conjugating them with the targeting ligand RCD and magnetic nanoparticles, followed by incubation with paclitaxel (PTX) to produce EV-PTX. The modified oncosomes exhibited enhanced cytotoxicity and demonstrated a significant tumor-targeting effect, highlighting their potential for pancreatic cancer therapy [52]. Although the incubation method offers several benefits, its major drawback is its relatively low drug-loading efficiency. As a result, it is commonly used in combination with other techniques to improve overall loading capacity [50].

***Transfection:*** a method that uses transfection reagents to introduce specific plasmids into donor cell vesicles, enabling the expression, encapsulation, and secretion of nucleic acid drugs [50]. A Yi Ba et al. study showed that delivering hepatocyte growth factor (HGF) siRNA via EVs can effectively inhibit the progression of gastric cancer. They transfected HEK293T cells with si.HGF-1 to load the siRNA into EVs, which were then isolated and applied as a treatment. These siRNA-containing EVs were taken up by gastric cancer cells, leading to a significant decrease in HGF and VEGF levels and resulting in reduced cell proliferation and migration. Also, animal studies demonstrated that treatment with siRNA. HGF-1-loaded EVs significantly lowered tumor size and blood vessel density [53]. Furthermore, Vakhshiteh et al. showed that MSCs transfected with miR-34a lentiviral vectors produced EVs enriched with miR-34a. These oncosomes were taken up by MDA-MB-231 breast cancer cells, leading to reduced expression of Bcl2 and c-MET, increased apoptosis (12.78%), and decreased migration (15%) and invasion (8.5%), highlighting that MSC-derived oncosomes could be used as carriers for miRNA-based cancer therapy [54]. While transfection provides high loading efficiency and molecular stability without requiring specialized equipment, it also has certain limitations. Some reagents may be toxic, raising safety concerns. Moreover, these reagents can contaminate exosomes, and differential centrifugation is not always used in separating transfection complexes from exosomes, which may impact the delivery of cargo to target cells [50].

***Electroporation:*** Electroporation is a technique that uses an external electric field near the membrane of vesicles; a short, high-voltage pulse temporarily disrupts the lipid bilayer, increasing its permeability. This allows therapeutic agents to enter the vesicles, which are then incubated to restore membrane integrity. This is one of the most efficient methods, which preserves the original properties of the drug and has superior loading efficiency compared to other techniques [50]. In the study by Liang et al., *5-fluorouracil (5-FU) and a miR-21 inhibitor (miR-21i) were loaded simultaneously into engineered EVs* via *electroporation* for targeted therapy against drug-resistant colorectal cancer cells (HCT-1165FR). This delivery system increased cancer cell uptake, downregulated miR-21 levels, and reactivated the tumor suppressors PTEN and hMSH2. When administered systemically in a mouse model, the dual-loaded exosomes significantly reduced tumor size and overcame resistance to 5-FU, showing greater efficacy than single-agent treatments [55]. Moreover, Mendt et al. developed clinical-grade engineered EVs (iEVs) derived from mesenchymal stem cells that were loaded with siRNA targeting the mutant Kras (G12D) via electroporation. These iEVs effectively reduced Kras expression in pancreatic ductal adenocarcinoma (PDAC) models, resulting in marked tumor shrinkage and increased survival inmouse models. It was found that iEVs have the potential to act as a safer therapy option for cancer-driven mutant Kras, owing to their non-immunogenic and non-toxic nature [56]. However, its effectiveness depends on multiple factors, including the number of EVs, the drug-to-vesicle ratio, the buffer type, the pulse capacitance, and the field strength [50].

***Sonication:*** Sonication is a method for loading therapeutic agents into oncosomes by applying mechanical shear via an ultrasound probe. This technique temporarily disrupts the vesicle membrane, enabling drug entry. It is also known for its high drug-loading efficiency and potential to enhance therapeutic effects [50]. For instance, Chen et al. proved that 5-FU-loaded oncosomes (5FO) using sonication significantly increased cytotoxicity in Cholangiocarcinoma cells (QBC93) growth, as compared to free 5-FU alone [57]. Additionally, Colja Alja Zottel et al. demonstrated that sonication effectively loaded the anti-vimentin nanobody Nb79 into glioblastoma-derived small extracellular vesicles (sEVs). This loading process resulted in a measurable reduction in vesicle diameter from approximately 180 nm to 140 nm while preserving membrane integrity. Importantly, the modified vesicles demonstrated biological activity by reducing the survival rate of NCH421k glioblastoma stem cells, thereby confirming the method’s effectiveness for therapeutic applications [58]. Although sonication improves drug loading efficiency and enables sustained drug release, it may also alter vesicle structure, affecting their shape, hydrophobic drug loading efficiency, and potentially causing aggregation. However, despite these challenges, it remains a widely used method for optimizing vesicle-based drug delivery [50].

***Freeze–thaw cycles:*** The freeze–thaw cycling method is a simple and efficient way for loading therapeutic drugs into vesicles. It involves repeatedly freezing and thawing the vesicle, which causes temporary ruptures and repairs, allowing drug incorporation while preserving membrane integrity and biological activity [50]. In a study, Khalid et al. used the freeze–thaw method to develop human-derived oncosomes loaded with hydroxyurea (Onco-HU). It was found that the developed Onco-HU showed pH-dependent, sustained drug release on MCF-7 breast cancer cell lines, along with showing superior cytotoxicity with an IC50 of 2.41 μM versus 91.19 μM for free hydroxyurea, indicating 30-fold increase in efficacy [59]. Ebrahimian et al. also used the freeze–thaw method for loading thymoquinone into EVs derived from MSCs. The developed oncosome reduced MCF-7 cell viability by 62% at doses equivalent to the IC50 value of free thymoquinone, demonstrating an efficient and safer delivery method for hydrophobic anticancer agents [60]. Although this method is suitable for large-scale production, it is less efficient than sonication and may lead to particle aggregation [50].

### Surface Modification for Targeting

Attaching specific ligands, such as peptides and antibodies, to the surface of oncosomes can enhance their ability to recognize and bind to specific targeted cancer cells, reducing toxicity to healthy cells. By using tumor-specific markers, researchers can engineer oncosomes that efficiently localize at the tumor site, increasing drug accumulation where it is needed the most [61]. Altering the genetic makeup of parent cells enables the production of oncosomes with modified surface proteins, thereby enhancing their targeting capabilities. This method involves engineering donor cells to express specific markers or receptors on oncosomes, facilitating improved interaction with target tumor cells [62].

## 7. Therapeutic Application

**Figure 6:**
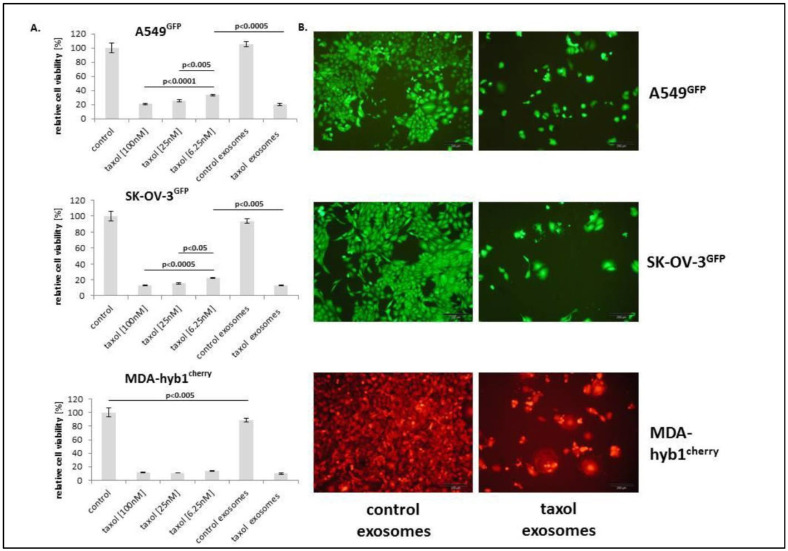
The chemotherapeutic sensitivity of various human cancer cell lines, A549GFP (lung cancer, top panel), SK-OV-3GFP (ovarian cancer, middle panel), and MDA-hyb1cherry (breast cancer, bottom panel), was assessed by evaluating relative cell viability after treatment with different concentrations of Taxol (100 nM, 25 nM, and 6.25 nM). These results were compared with those of the corresponding cancer cells maintained at the highest Taxol concentration, which served as the control. Additionally, the cytotoxic potential of two types of exosomes was analyzed: control exosomes (isolated from MSCs cultured in the highest concentration of the taxol solvent) and taxol-loaded exosomes (1:150 dilution, derived from MSCs treated with 10 µM taxol). After a 72 h incubation period, cell viability was determined using fluoroscan assays, performed in triplicate (**A**). Results are presented as mean ± standard deviation (s.d.), with controls normalized to 100%. Statistical analysis was carried out using ANOVA followed by Tukey’s post hoc test. Fluorescence microscopy was employed to observe morphological changes in the cancer cell lines after 72 h of treatment. Images on the left show cells incubated with control exosomes, while those on the right display the effects of taxol-loaded exosomes (**B**). Scale bars represent 200 µm. Reproduced with permission from [63].

**Figure 7:**
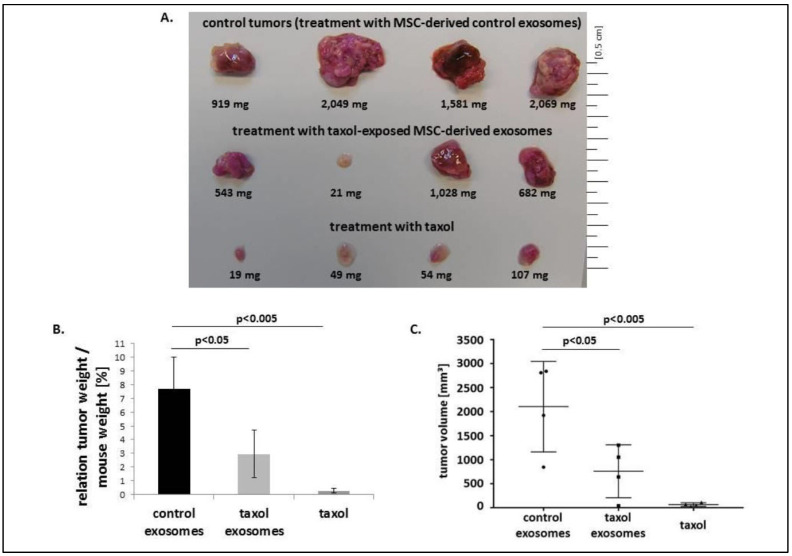
Human mCherry-labeled MDA-hyb1 breast cancer cells were injected into NODscid mice to develop tumors. (**A**) The weight of the tumors in each treatment group (n = 4) was recorded following the dissection of the primary subcutaneous tumors (excluding any metastatic tumor tissue). (**B**) The mice’s body weight was also measured, and the tumor-to-body weight ratio was computed. Data are presented as the mean ± standard deviation, and statistical significance (*p*) was assessed using ANOVA followed by Dunnett’s multiple comparisons test. (**C**) Tumor length and width were measured, and the average tumor volume for each treatment group was calculated using the formula in the reference. Data are presented as mean ± standard deviation, and statistical significance (*p*) was assessed using ANOVA followed by Dunnett’s multiple comparisons test. Reproduced with permission from [63].

**Figure 8:**
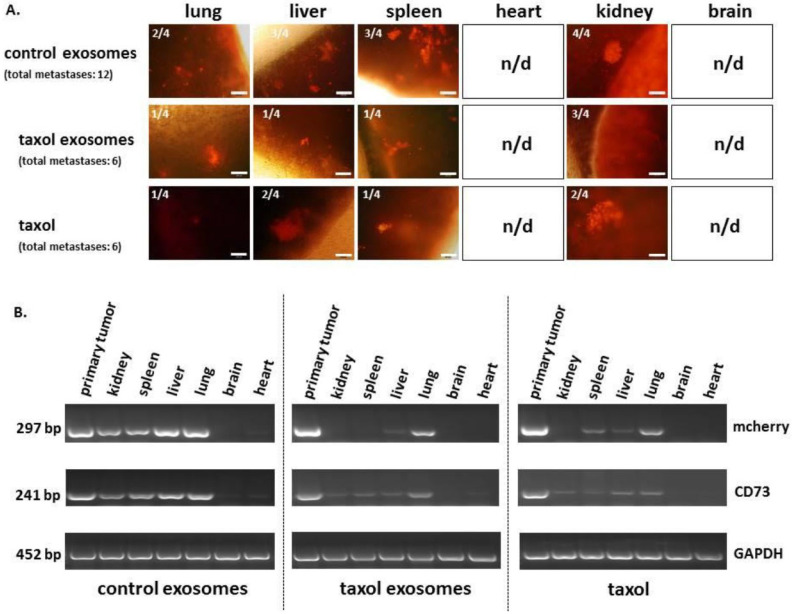
The presence of distant organ metastases was identified by the appearance of mCherry fluorescence in thin sections of organ tissues. Representative phase contrast/fluorescence microscopy overlay images of organ metastases are shown. Additionally, the total number of organs containing metastatic tumor cells is presented (n.d. = not detectable). Scale bars represent 100 μm (**A**). PCR analysis of both primary tumor and organ tissue samples was conducted to confirm the presence of metastatic MDA-hyb1 cancer cells, indicated by the detection of mCherry and CD73 transcripts (**B**). Reproduced with permission from [63].

Breast cancer stands as the most prevalent cancer diagnosed globally and is a major contributor to cancer-related death in women [1]. Although current treatment options, such as surgery, chemotherapy, and radiation, have improved survival rates, their effectiveness is often compromised by drug resistance, limited targeting ability, and significant side effects. Paclitaxel (Taxel) is a standard chemotherapeutic agent used in breast cancer therapy due to its ability to stabilize microtubules and induce apoptosis. However, its therapeutic potential is limited by poor water solubility and nonspecific toxicity. To address these challenges, Melzer et al. explored an innovative delivery strategy using EVs derived from MSCs. Their study demonstrated that Taxol-EVs (TEVs) reduced the viability and proliferation of MDA-hyb1 breast cancer cells, as measured by fluoroscan assays and fluorescence microscopy (Figure 6). Notably, the exosomal delivery system achieved this cytotoxic effect with ~1000-fold less Taxol than required with the free drug. In vivo, TEVs reduced tumor weight by more than 60% and decreased the number of distant organ metastases by 50% compared with untreated controls (Figure 7 and Figure 8). Additionally, tumor-targeted delivery of TEVs resulted in 34-fold higher accumulation at tumor sites relative to systemic distribution, highlighting their potential to be used as a highly efficient, low toxicity platform for breast cancer chemotherapy [63].

**Figure 9:**
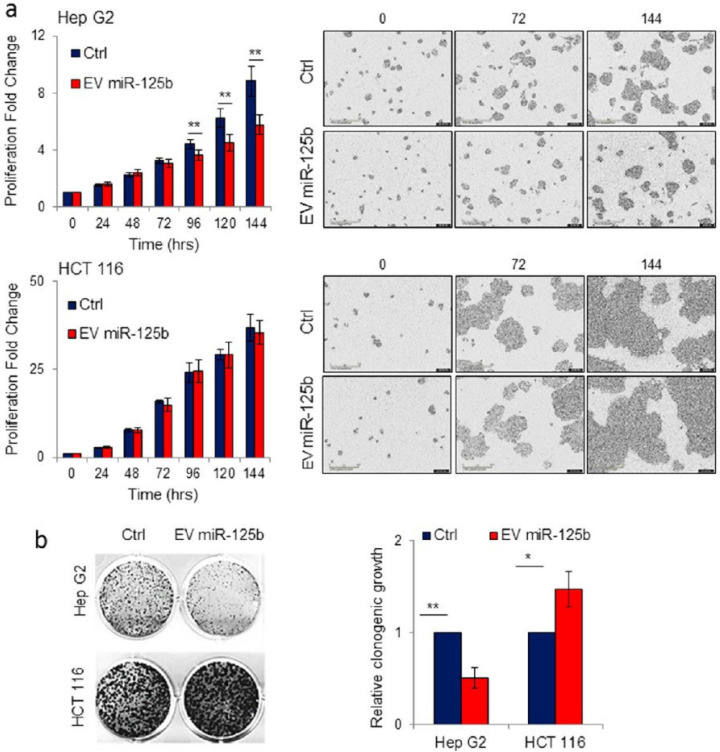
Delivery of Engineered EVs Containing miR-125b from Adipose-Derived Stromal Cells reduces HCC Proliferation. (**a**) Human liver cancer (Hep G2) and colorectal cancer (HCT 116) cell lines were exposed to 10 µg of EVs obtained from adipose-derived stromal cells that had been genetically modified to express ExoMotif-tagged miR-125b or a control vector. Cell proliferation was tracked over time using the IncuCyte^®^ live-cell imaging system, with representative images captured at defined intervals using a 10× objective lens. (**b**) To assess long-term effects, a colony formation assay was performed on both HepG2 and HCT116 cells following EV treatment. After a seven-day incubation period, colonies were visualized with crystal violet staining. Quantification of colony growth was performed by densitometric analysis of the stained cultures. Reproduced with permission from [64]. * *p* < 0.05, ** *p* < 0.01.

In contrast, hepatocellular carcinoma (HCC) ranks among the most frequently diagnosed types of cancer after breast cancer, along with being a major contributor to deaths related to cancer. Although surgical removal of tumors is considered the most acute treatment, only a small proportion of patients qualify for this option due to the financial burden [1]. As a result, there is a demand to identify novel therapeutic methods for targeted therapy that interfere with molecular mechanisms driving tumor development or upregulate tumor suppressor function. In HCC, several miRNAs exhibit abnormal expression patterns associated with disease progression. One such molecule is miR-125b, which is commonly found at reduced levels in patients with HCC and in cancer cell models. Restoring miR-125b levels in HCC cells has been shown to inhibit cancer cell growth and metastasis by disrupting the activity of key oncogenes, including LIN28B, Mcl-1, IL6R, and others, underscoring its potential for HCC treatment. Baldari et al. *studied the effects of miR-125b-loaded* EVs in HCC cell lines. The findings from live-cell imaging (IncuCyte) and WST-1/colony formation assay reveal inhibition of cell proliferation in HepG2 and no effect in colon cancer HCT 116 cells (Figure 9) [64].

**Figure 10:**
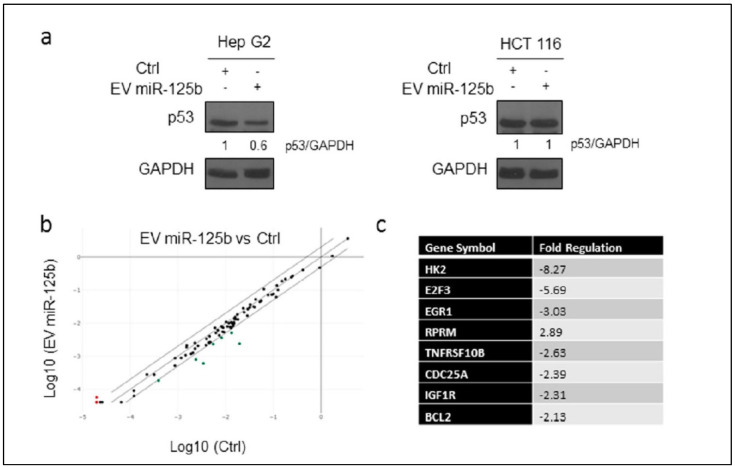
Delivery of EVs isolated from adipose tissue derived from stromal cells genetically modified with ExoMotif-tagged microRNA-125b affecting the p53 expression. (**a**) Immunoblot analysis assessed p53 protein expression in HepG2 and HCT 116 cells after 144 h of treatment with extracellular vesicles (EVs) either enriched with ExoMotif-tagged miR-125b or from control cells. GAPDH served as the internal loading reference. Densitometry analysis performed using ImageJ software is indicated. (**b**) To explore the broader impact on the p53 signaling network, a targeted RT2 Profiler PCR Array was utilized, profiling 84 genes associated with the p53 pathway in both miR-125b EV-treated and control groups. A scatter plot illustrated differential gene expression, with red and green markers indicating significantly upregulated and downregulated genes, respectively. (**c**) A summary table highlighted genes that exhibited at least twofold changes in expression in response to miR-125b-loaded EV treatment compared with the control group. Reproduced with permission from [64].

Also, it was found that EVs induced downregulation of p53 protein along with other genes in p53 signaling pathway such as BCL2, IGF1, HK1, and E2F3, while upregulating tumor suppressor gene RPRM only in HepG2 cell line, showing good targeting ability of miR-125b, confirmed by specific RT2 Profiler PCR Array (Figure 10) [64].

**Figure 11:**
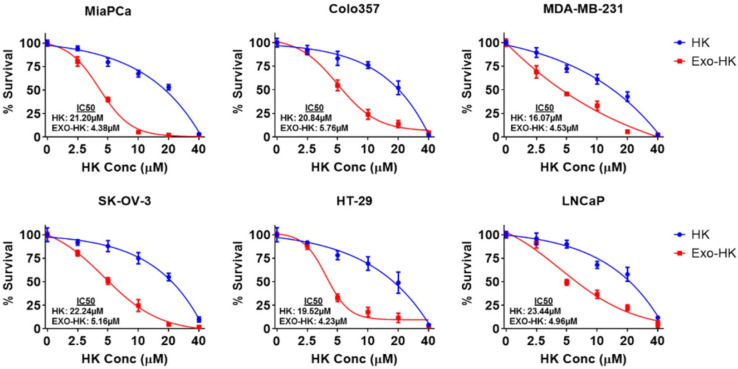
The cytotoxic potential of exosome-encapsulated honokiol was evaluated in multiple human cancer cell lines, including pancreatic (MiaPaCa, Colo357), breast (MDA-MB-231), ovarian (SK-OV-3), colon (HT-29), and prostate (LNCaP) cells. Each cell line was seeded at 5 × 10^3^ cells per well in 96-well plates and treated for 72 h with vehicle control, unloaded exosomes, free honokiol, or exosomal honokiol at concentrations ranging from 0 to 40 μM. Cell viability was assessed using the WST-1 assay, and data were reported as mean ± standard deviation (n = 3), normalized to respective controls. A statistically significant reduction in cell viability (*p* < 0.05) was observed across the 5–20 μM range, with the exosomal formulation demonstrating greater cytotoxicity than the free drug. Reproduced with permission from [65].

**Figure 12:**
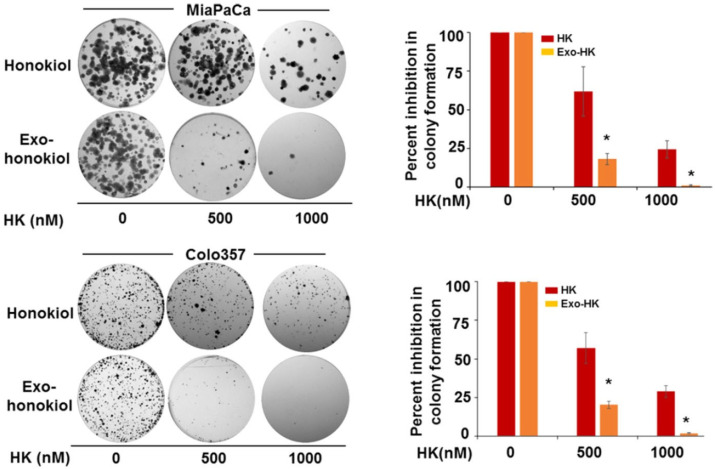
The clonogenic assay was conducted to evaluate the effect of exosome-encapsulated honokiol on the long-term proliferative ability of pancreatic cancer cells. MiaPaCa and Colo357 cells were seeded into six-well plates at a density of 1 × 10^3^ cells per well. After a 24 h attachment period, the cells were treated with either free honokiol or its exosomal form (exo-HK) at 0, 500, or 1000 nM. Following two weeks of incubation to allow colony formation, the cells were fixed, stained, and imaged. Colony counts were performed using Gene Tools image analysis software (Syngene, Frederick, MD). Data were expressed as the percentage of clonogenic inhibition relative to untreated controls and presented as the mean ± standard deviation (n = 3). A significant reduction in colony formation was observed in cells treated with exosomal honokiol compared to those treated with free honokiol (* p < 0.05). Reproduced with permission from [65].

Furthermore, to enhance the clinical potential of honokiol, a natural anticancer agent with poor bioavailability, Ajay Pratap Singh et al. prepared an exosomal formulation using MSC-derived exosomes. Using six sonication cycles and a 1:4 honokiol-to-exosome ratio, they achieved approximately 80% drug loading, with particles averaging 171.7 nm in diameter and a zeta potential of −12.5 mV. The formulation demonstrated 4–5 times greater anticancer potency compared to free honokiol across multiple cancer cell lines (Figure 11) and significantly suppressed colony formation in pancreatic cancer models (Figure 12). Additionally, there was a 3.64-fold and 4.68-fold increase in intracellular accumulation of honokiol in MiaPaCa and Colo357, respectively, indicating improved cellular delivery [65].

## 8. Clinical Trials and Insights

EV’s potential, numerous early-phase clinical trials have been conducted to investigate the use of oncosomes as therapeutic cargo carriers. For instance, a phase 1 clinical trial conducted by Brandon G. Smagl et al. revealed that the EVs loaded with siKRAS^G12D were able to control cancer progression by decreasing the KRAS^G12D level, with 28.8 mg of protein being the best dose, in patients with pancreatic cancer, without any severe side effects [66]. Additionally, two clinical trials (NCT04879810 and NCT01294072) are still under investigation, exploring the use of curcumin-loaded EVs as a potential treatment for colon cancer and irritable bowel disease (IBD) [67]. Although no efficient outcomes have been reported yet, these trials set a precedent for EV-based therapeutics.

## 9. Challenges in the Development of Oncosomes

Despite oncosome’s potential promise in conceptual relevance to precision oncology as vehicles for targeted drug delivery, several scientific and technical barriers continue to impede their clinical advancement. A critical issue is the inherent heterogeneity of EVs, which differ considerably in size, surface biomarkers, and cargo composition. This variability complicates efforts to isolate a homogeneous population that is reproducible and sustainable for therapeutic use [68]. The lack of uniformity impedes the standardization of protocols and undermines the reliability of preclinical and clinical outcomes. Additionally, existing isolation and purification strategies are often inefficient and can damage vesicle structural integrity. These techniques may yield low levels and lead to co-isolation of contaminants, thereby limiting the purity and scalability of oncosome production [69]. Another significant challenge is the low efficiency and inconsistent loading of therapeutic cargo. Common methods such as electroporation, freeze–thaw cycling, and sonication may achieve sufficient encapsulation of bioactive molecules but, in some cases, may also cause vesicle deformation or loss of function [70]. Moreover, the scale-up of production to meet clinical and industrial demands remains unresolved. Achieving reproducible, GMP-compliant large-scale manufacturing while maintaining biological activity and product safety is technically demanding and cost-intensive. In addition to production issues, maintaining physical and biological stability during storage and transportation is another pressing concern. Oncosomes are prone to aggregation, degradation, or cargo loss, all of which can impair their therapeutic efficacy and shelf life [71]. Finally, there are regulatory and safety concerns, especially when using tumor-derived vesicles. These vesicles may contain oncogenic factors or activate unwanted oncogenic pathways and immune responses, as they are normally released by cancer cells, raising questions about their biocompatibility and long-term safety [72,73]. Also, compared with other well-established nanocarriers, such as exosomes and microvesicles, there is limited clinical data available. Furthermore, the field currently lacks standardized protocols for production, quality control, and regulatory approval pathways [74]. Altogether, while the therapeutic potential of oncosomes is compelling, addressing these multifaceted challenges will require technological innovation, regulatory harmonization, and interdisciplinary collaboration. Importantly, the majority of evidence supporting oncosome-based drug delivery remains preclinical, derived largely from in vitro studies and animal models that demonstrate proof of concept rather than therapeutic efficacy. While these studies highlight mechanistic feasibility and biological relevance, they should not be interpreted as indicators of clinical readiness. To date, clinical investigations are limited and primarily focused on safety, feasibility, or biomarker exploration, underscoring the early developmental stage of oncosome-based therapeutics.

## 10. Regulatory Requirements

Currently, approval of EV-based therapies varies across regions. In the United States, the Food and Drug Administration (FDA) oversees these products as drugs and biologics under the Public Health Service (PHS) Act and the Federal Food, Drug, and Cosmetic Act (FD&C Act), requiring compliance with cGMP and complete premarket evaluation [75]. In the European Union, EVs without functional RNA are treated as biological medicines, while EVs carrying active RNA having therapeutic effects are regulated as advanced therapy medicinal products (ATMPs) [76]. Japan regulates EVs derived from non-living cells as biologics, applying the same standards as for vaccines and blood products, with approval requiring clinical trials and review by the Pharmaceutical and Medical Devices Agency (PMDA) under the Ministry of Health, Labour and Welfare (MHLW). Across all regions, common requirements include GMP compliance, manufacturing, validated quality control, batch-to-batch consistency, and a clear understanding of the mechanism of action (MOA). The proper regulatory alignment for EV is still limited due to the absence of standardized potency tests, and incomplete clinical safety and efficacy [77,78,79]. Though regulatory challenges exist, safety concerns include oncogenic cargo, tumor progression, long-term biodistribution risks, and immune modulation. This interpretation is required for further safety evaluation.

## 11. Future of Oncosome Therapies

Drug or genetically loaded oncosome showcases that it may warrant further investigation as a potential therapeutic strategy [80]. These tiny EVs have shown tumor-associated uptake in preclinical models, allowing them to deliver medicine directly to the cancer site, which increases treatment efficacy and reduces side effects on healthy tissue [81,82]. They can also carry molecules that silence genes or block pathways that cancer cells use to resist drugs, helping in treatment resistance [83,84]. Additionally, oncosomes can be used for delivering multiple drugs to the same target, allowing combined therapies that attack cancer cells [85]. Because oncosomes can also be derived from the patient’s tumor cells, they may be tailored to specific tumor characteristics, supporting personalized medicine approaches. While there are still challenges associated with oncosome production and loading efficiency, and limited clinical data, research is progressing rapidly to overcome these issues and advance translational research toward potential clinical evaluation.

## 12. Conclusions

Due to their natural compatibility with the human body, their ability to act as a protective barrier for therapeutic agents, and their inherent targeting capabilities for precise delivery to tumor cells, oncosomes offer a significant approach to cancer treatment that could help overcome the drawbacks of conventional therapies. Additionally, advancements in drug-loading methods, including sonication, electroporation, and transfection, have improved the efficiency of encapsulating various therapeutic molecules, such as chemotherapeutic agents and nucleic acids. Moreover, research has also demonstrated the effectiveness of EV therapies across diverse cancer types. For example, extracellular vesicles loaded with paclitaxel have significantly inhibited breast cancer progression, while miR-125 b-enriched vesicles have reduced liver cancer cell growth by modulating critical molecular pathways. Similarly, EV-encapsulated honokiol has shown superior anticancer activity and increased intracellular drug accumulation compared to free honokiol, highlighting its potential in pancreatic and other cancers. These findings highlight the versatility and potential of EV-based platforms to improve drug stability, targeting accuracy, and therapeutic outcomes in oncology. Continued efforts to optimize large-scale production, enhance targeting specificity, and conduct clinical trials will be essential to fully realize their application in personalized cancer therapy.

## Figures and Tables

**Table 1:** Molecular components of large oncosomes (LOs) and their functions.

Category	Molecules	Function/Role in Cancer Progression	Reference
Proteins	Glutaminase (GLS)	Converts glutamine to glutamate. Promotes metabolic reprogramming and supports the Warburg effect.	[23]
	Glyceraldehyde 3-phosphate dehydrogenase (GAPDH)	An enzyme in glycolysis. Contributes to altered cancer cell metabolism	
	Malate dehydrogenase (MDH)	Participates in the citric acid cycle. Supports energy production in tumor cells	
	Cytokeratin 18 (CK18)	Structural protein. Potential marker for tumor-derived EVs	[24]
	Heat shock protein 70 (HSPA5)	Aids protein folding. Involved in stress response and tumor cell survival.	
Nucleic acids	miRNAs	Regulates gene expression in the recipient cell. Certain miRNAs in Los stimulate cancer-associated fibroblast migration and promote tumor progression.	[25]
	mRNAs	mRNAs within LOs may be translated in recipient cells. Alters cellular phenotype and supports tumor growth.	
Sphingolipids	Sphingomyelin, ceramides	Increase membrane rigidity. Promotes the formation of lipid rafts, which are essential for vesicle stability and signaling.	[26,27,28]
Glycosphingolipids	Ganglioside GM3	Mediates cell–cell interactions. Modulates tumor-microenvironment communication.	
Sterols	Cholesterol	Maintains membrane integrity. Supports raft-mediated oncogenic signaling.	[28]
	Cholesteryl esters	Correlates with metastatic potential, especially in prostate cancer	
phospholipids	Phosphatidylserine (PS)	Externalized on the LO surface. Promotes uptake of recipient cells and may aid immune evasion.	[29]
	Phosphatidylethanolamine (PE), phosphatidylcholine (PC)	Contributes to membrane fluidity and structural dynamics.	[27]
Ether-linked lipids	Plasmalogens (notably in colorectal cancer-derived LOs)	Improve membrane fluidity and vesicle fusion to enhance the delivery of oncogenic cargo.	[26]

**Table 2:** Characteristics and Functions of Oncosomes.

Feature	Details	Reference
Size	100–400 nm (typical); Large Oncosomes (LOs): 1–10 μm	[18]
Origin	Blebbing off the plasma membrane of non-apoptotic tumor cells	[22]
Triggering factors	Cell transformation, EGFR/AKT1 pathway activation, cytoskeletal remodeling, calcium influx	[18,22]
Markers	High: Cytokeratin-18; Low: CD9, CD63, CD81	[20]
Structural proteins involved	Caveolin-1 (CAV-1), HB-EGF, MyrAkt1, ARF6, DIAPH3	[17]
Contents	Metalloproteinases, proteins, mRNAs, miRNAs, lipids, bioactive molecules	[23,28,34]
Unique features	Amoeboid migration triggers gigantic EVs, Abnormal cargo loading	[35]
Biological roles	Tumour growth, ECM degradation, angiogenesis, immune suppression, endothelial permeability, metastatic colonization	[19]
Target cells	Tumor cells, stromal cells, endothelial cells	

**Table 3:** Outcome of various strategies.

Cargo		Oncosome Source	Loading Technique	Targeted Cell Lines	Outcomes	Reference
Doxorubicin		Adipose mesenchymal stem cells	Sonication	Breast cancer	Decrease in IC50 value Downregulation of H19 and UCA1 IncRNAs. Upregulation of TP53 expression. Strong tumor suppression in vivo.	[44]
		Platelet extracellular vesicles	Freeze/thaw		Targeted delivery to breast cancer cells. Increase sensitivity in drug-resistant cell lines.	[45]
Paclitaxel		LNCap PC-3	Incubation	Prostate cancer	Enhance the cytotoxic effect	[46]
Nucleic acid	miR-449a	Engineered exosomes (miR-449a)	Transfection	Lung cancer	Decrease in cell viability. Reduced expression of Bcl-2. Increased cell apoptosis. Decrease in migration and invasion ability. Showed anti-tumor effect in in vivo.	[47]
	miRNA-21	HEK293T cell	Electroporation	Brain tumor	Passed through the BBB. Suppression of tumor growth.	[48]
Protein	SIRP ∝	HEK293T cell	Transfection	Colon carcinoma	Enhanced phagocytosis of tumor cells. Suppression of tumor growth.	[49]
	T34A	Melanoma cell	Transfection	Pancreatic Adeno carcinoma	Induced apoptosis in adenocarcinoma cells. Enhanced drug sensitivity.	[49]

## Data Availability

No new data generated.

## Abbreviations

WHO: World Health Organisation; GLOBOCAN: The Global Cancer Observatory; DDS: Drug-delivery system; EVs: Extracellular vesicles; EGFRvIII: Epidermal growth factor receptor variant III; siRNA: Small interfering RNAs; MVBs: Multivesicular bodies; RNA: Ribonucleoside; MVs: Microvesicles; MMPs: Matrix metalloproteinases; DIAPH3: Diaphanous-related formin-3; CAV-1: Caveolin-1; HB-EGF: Heparin-binding epidermal growth factor; MyrAkt1: Myristoylated Akt1; EGFR: Epidermal growth factor receptor; GLS: Glutaminase; GAPDH: Glyceraldehyde 3-phosphate dehydrogenase; CK18: Cytokeratin 18; HSPA5: Heat shock protein 70; PS: Phosphatidylserine; PE: Phosphatidylethanolamine; PC: Phosphatidylcholine; TDEs: Tumour-derived exosomes; ITGβ4: Integrin β4; Los: Large Oncosomes; HGF: hepatocyte growth factor
